# Less invasive replacement of aortic root, ascending aorta and hemiarch via partial upper sternotomy: a propensity-score-matched comparison with full sternotomy

**DOI:** 10.1093/icvts/ivae120

**Published:** 2024-06-28

**Authors:** Nestoras Papadopoulos, Vasileios Ntinopoulos, Achim Haeussler, Dragan Odavic, Petar Risteski, Héctor Rodríguez Cetina Biefer, Omer Dzemali

**Affiliations:** Department of Cardiac Surgery, Municipal Hospital of Zurich, Zurich, Switzerland; Department of Cardiac Surgery, University Hospital of Zurich, Zurich, Switzerland; Department of Cardiac Surgery, Municipal Hospital of Zurich, Zurich, Switzerland; Department of Cardiac Surgery, University Hospital of Zurich, Zurich, Switzerland; Department of Cardiac Surgery, Municipal Hospital of Zurich, Zurich, Switzerland; Department of Cardiac Surgery, University Hospital of Zurich, Zurich, Switzerland; Department of Cardiac Surgery, Municipal Hospital of Zurich, Zurich, Switzerland; Department of Cardiac Surgery, University Hospital of Zurich, Zurich, Switzerland; Department of Cardiac Surgery, Municipal Hospital of Zurich, Zurich, Switzerland; Department of Cardiac Surgery, University Hospital of Zurich, Zurich, Switzerland; Department of Cardiac Surgery, Municipal Hospital of Zurich, Zurich, Switzerland; Department of Cardiac Surgery, University Hospital of Zurich, Zurich, Switzerland; Department of Cardiology, Center for Translational and Experimental Cardiology (CTEC), University Hospital of Zurich, University of Zurich, Zurich, Switzerland; Department of Cardiac Surgery, Municipal Hospital of Zurich, Zurich, Switzerland; Department of Cardiac Surgery, University Hospital of Zurich, Zurich, Switzerland; Department of Cardiology, Center for Translational and Experimental Cardiology (CTEC), University Hospital of Zurich, University of Zurich, Zurich, Switzerland

**Keywords:** Less invasive surgery, Partial upper sternotomy, Aortic root, Ascending aorta, Hemiarch, Proximal aortic arch, Concomitant surgery

## Abstract

**OBJECTIVES:**

Less invasive surgery has emerged as an option for aortic pathologies. The current study compared our experience on early postoperative results of patients with aortic surgery between partial upper sternotomy (PUS) and full sternotomy (FS).

**METHODS:**

We performed a retrospective analysis of the data of patients undergoing aortic root surgery with concomitant ascending aorta and hemiarch replacement. Exclusion criteria were type A aortic dissection and other concomitant major cardiac surgery. After propensity score matching, we compared the perioperative outcomes of patients undergoing surgery with PUS versus FS.

**RESULTS:**

A total of 161 patients operated on between January 2013 and September 2022 met the inclusion criteria (PUS: *n* = 22, FS: *n* = 139). Propensity score matching yielded 22 pairs with a balanced distribution of propensity scores and covariates between the compared groups. There was no evidence that PUS affects cardiopulmonary bypass [108 (67–119) vs 113 (87–148) min, *P* = 0.154; PUS vs FS] and circulatory arrest duration [9 (7–10) vs 9 (8–13) min, *P* = 0.264; PUS vs FS]. There was a reduced cross-clamp duration in the PUS group [88 (58–96) vs 92 (71–122) min, *P* = 0.032]. Cumulative sum charts have shown consistently low cross-clamp and circulatory arrest duration for 2 experienced surgeons who performed 20 of the procedures in the PUS group (10 each). Perioperative mortality and morbidity were low, with no in-hospital mortality in the PUS group [0 vs 1(4.5%), *P* > 0.999] and absence of strokes in both groups.

**CONCLUSIONS:**

In summary, our initial experience suggests that less invasive aortic root, ascending aorta and hemiarch replacement via PUS could be performed in our patient cohort as safely as via full sternotomy. Advantages for the patient are reduced surgical trauma, improved cosmetic results and—presumably—less pain.

## Graphical Abstract

**Figure:**
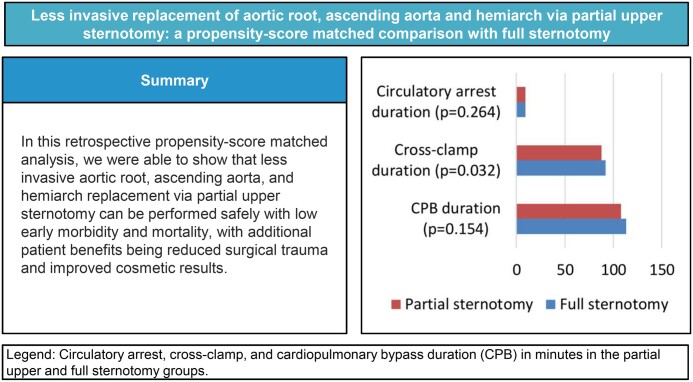

## INTRODUCTION

Full sternotomy (FS) represents the gold standard for patients undergoing aortic root surgery with concomitant ascending aorta and hemiarch replacement [1, 2]. Occasional series reported the feasibility of performing isolated aortic root or ascending aorta and hemiarch replacement via less invasive access such as partial upper sternotomy (PUS) [3–5].

Less invasive approaches are at the forefront of contemporary cardiac surgery mainly due to accumulating evidence for favourable outcomes like reduction of pain, ventilation time and transfusion requirement, as well as higher patient acceptance and improved cosmetic results [6–8].

Despite the clear advantages of minimally invasive surgery, scarce evidence regarding the safety and perioperative outcome of less invasive aortic root surgery with a concomitant ascending aorta and hemiarch replacement through PUS remains.

The current study compared the early postoperative results of patients undergoing aortic root surgery with concomitant ascending aorta and hemiarch replacement via PUS versus FS, using propensity-score-matched analysis. Furthermore, an assessment of our learning curve, using cumulative sum (CUSUM) charts, has been performed.

## PATIENTS AND METHODS

### Ethical statement

The study was approved, and individual informed consent was waived by the local ethics committee, on 5 September 2023 (Ethics Committee of the Canton of Zurich, Business Administration System for Ethics Committees-Number: 2023-00393).

### Study design

We performed a retrospective, single-centre (Municipal Hospital of Zurich) analysis of the data of patients undergoing aortic root, ascending aorta and hemiarch replacement through PUS or FS between January 2013 and September 2022. We hypothesized that aortic root surgery with concomitant ascending aorta and hemiarch replacement performed via a less invasive approach (PUS) was not inferior regarding perioperative mortality and morbidity compared to FS. We excluded patients with acute or chronic aortic dissection and those undergoing other concomitant major cardiac surgery; the following concomitant cardiac procedures were, however, included: bipolar pulmonary vein isolation, left atrial appendage occlusion, patent foramen ovale closure and subaortic septal myomectomy. Due to changes in our practice, we are currently employing antegrade selective cerebral perfusion during circulatory arrest. Patients undergoing circulatory arrest without antegrade selective cerebral perfusion were excluded to avoid bias. Table 1 summarizes patient data that were collected in the current study.

**Table 1:** Preoperative characteristics of the study population

	Unmatched patients, *n* = 161			Matched patients, *n* = 44		
	Full sternotomy, *n* = 139	Partial sternotomy, *n* = 22	SD	Full sternotomy, *n* = 22	Partial sternotomy, *n* = 22	SD
Age (years)	60 (52–70)	60 (53–68)	0.02	50 (46–61)	60 (53–68)	0.542
Female gender	32 (23)	4 (18.2)	0.12	5 (22.7)	4 (18.2)	0.113
BMI (kg/m^2^)	27.3 (4.7)	26.8 (3.7)	0.117	26.4 (23.6–29.7)	26.4 (24.6–29.1)	0.069
NYHA class 3 or more	27 (19.4)	2 (9.1)	0.299	2 (9.1)	2 (9.1)	0
Arterial hypertension	88 (63.8)	13 (59.1)	0.096	13 (61.9)	13 (59.1)	0.058
Dyslipidaemia	61 (44.2)	8 (36.4)	0.16	7 (33.3)	8 (36.4)	0.064
Diabetes mellitus	13 (9.4)	3 (13.6)	0.135	3 (13.6)	3 (13.6)	0
Current or previous smoker	66 (48.2)	12 (57.1)	0.18	13 (61.9)	12 (57.1)	0.097
LVEF (%)	60 (55–65)	60 (58–65)	0.044	60 (55–61)	60 (58–65)	0.194
Blood creatinine (mmol/l)	78 (69–91)	79 (73–96)	0.002	79 (67–91)	79 (73–96)	0.204
COPD	7 (5)	1 (4.5)	0.023	1 (4.5)	1 (4.5)	0
Previous cerebrovascular insult	8 (5.8)	1 (4.5)	0.057	0	1 (4.5)	0.309
Peripheral arterial disease	3 (2.2)	0	0.21	0	0	0
Acute endocarditis	5 (3.6)	0	0.275	0	0	0
Previous PCI	6 (4.3)	2 (9.1)	0.192	2 (9.1)	2 (9.1)	0
Previous cardiac surgery	10 (7.2)	1 (4.5)	0.113	2 (9.1)	1 (4.5)	0.181
Non-elective surgery	8 (5.8)	0	0.349	0	0	0
EuroSCORE II (%)	2.7 (2–4.5)	2.3 (1.8–3)	0.16	2.2 (1.6–2.8)	2.3 (1.8–3)	0.147

Continuous variables are reported as mean (standard deviation) or median (interquartile range), and categorical variables as counts and percentages, *n* (%).

BMI: body mass index; COPD: chronic obstructive pulmonary disease; EuroSCORE: European System for Cardiac Operative Risk Evaluation; LVEF: left ventricular ejection fraction; NYHA: New York Heart Association; PCI: percutaneous coronary intervention; SD: standardized difference.

### Surgical technique

Less invasive procedures were performed through left-sided L-shaped PUS with the incision beginning at the manubrium sterni and ending at the left 4th intercostal space. The remaining procedures were performed via FS. Arterial cannulation for the cardiopulmonary bypass was performed with direct cannulation of the right subclavian artery. Venous cannulation was performed either through the right atrium or, in some cases, of PUS with minimal surgical view percutaneously through the right femoral vein. Left ventricular venting was performed by placing a cannula through the right superior pulmonary vein or the roof of the left atrium. Hemiarch replacement was performed under circulatory arrest and antegrade selective cerebral perfusion over the right subclavian artery. Circulatory arrest was performed with deep hypothermia (≤20°C) in 1 patient, moderate hypothermia (20.1–28°C) in 33 patients, mild hypothermia (28.1–34°C) in 115 patients and normothermia (>34°C) in 12 patients, with a trend for reducing the grade of hypothermia over the study period. The Bretschneider cardioplegic solution was used for myocardial protection and was applied indirectly antegrade to the ascending aorta or the coronary ostia in case of severe aortic regurgitation. If necessary, cardioplegia was repeated after 120 min of cardioplegic arrest, allowing an ischaemia time of 100–120 min.

Expected challenges for the PUS approach to become a standard procedure, including young surgeons, represent, on the one hand, the dissection of the brachiocephalic trunk and left carotid artery as preparation for isolated antegrade cerebral perfusion and, on the other hand, the approach of the non-coronary sinus. Deep right-sided pericardial stitches are recommended to improve the exposure of the above-mentioned anatomical regions. Apnoea is a crucial safety precaution to avoid lung injury and a safety distance from the right-sided phrenic nerve. Latero-caudal traction on these sutures allows better exposure of the brachiocephalic trunk, ascending aorta and non-coronary sinus. Furthermore, dissection of the brachiocephalic trunk and left carotid artery can be facilitated on cardiopulmonary bypass before cross-clamping the ascending aorta.

### Data analysis

The statistical analyses were performed with IBM SPSS Statistics for Windows, Version 27.0 (IBM, Armonk, NY, USA) and R (R Foundation for Statistical Computing, Vienna, Austria). We performed a between-group comparison of the preoperative, intraoperative and postoperative characteristics of patients with PUS versus FS before and after propensity score matching. Propensity scores were calculated with logistic regression, using the binary partial/full-sternotomy variable as a dependent variable and the following as predictor variables: age ≥65 years, body mass index ≥30 kg/m^2^, left ventricular ejection fraction ≤35%, high preoperative creatinine values (≥84 mmol/l for females and ≥104 mmol/l for males), European System for Cardiac Operative Risk Evaluation II (EuroSCORE II) ≥4%, female gender, New York Heart Association (NYHA) class ≥3, diabetes mellitus, chronic obstructive pulmonary disease, previous cerebrovascular insult (CVI), peripheral arterial disease, acute endocarditis, previous percutaneous coronary intervention, previous cardiac surgery and non-elective surgery. Propensity score matching was performed one-to-one (1:1), with the nearest-neighbour-method, using the ‘pairmatch’ function of the ‘optmatch’ package in R. Balance of individual covariates across treatment and comparison groups was controlled through computation of the standardized differences of the individual covariates before and after propensity score matching. A learning curve assessment evaluating the aortic cross-clamping duration and circulatory arrest duration over time was performed with CUSUM charts similarly to our previously published work [9]. For the CUSUM charts, the target value was set at the centre of group statistics (for each surgeon), the decision interval at 5 standard errors and the shift detection at 1 standard error. The CUSUM analysis was performed with the ‘qpc’ package in R. Primary outcomes of interest were death, CVI (stroke), rethoracotomy for bleeding or tamponade and postoperative stay. Secondary outcomes of interest were renal replacement therapy, intubation duration, intensive care unit stay, blood product transfusion, myocardial infarction and echocardiographic parameters (left ventricular ejection fraction, mean and peak aortic valve gradient, aortic valve regurgitation).

Categorical variables are presented as counts (percentages), continuous as mean (standard deviation) by normally distributed data and median (interquartile range) by non-normally distributed data. The normality of data distribution was assessed mainly using Q–Q plot and histogram inspection and secondarily with the Shapiro–Wilk and the Kolmogorov–Smirnov test. Continuous data were compared with the Student’s *t*-test or the Mann–Whitney *U*-test according to the normality of data distribution. Categorical data were compared with the chi-squared or Fisher’s exact test according to the number of cells with an expected count of less than 5 in the respective contingency tables. Paired tests were used in the matched sample (paired Student’s *t*-test or Wilcoxon signed-rank test for continuous variables, and McNemar’s test for categorical variables). All tests were two-sided, and the level of statistical significance was set at 0.05. Cases with missing data were handled with pairwise deletion.

## RESULTS

**Figure 1:**
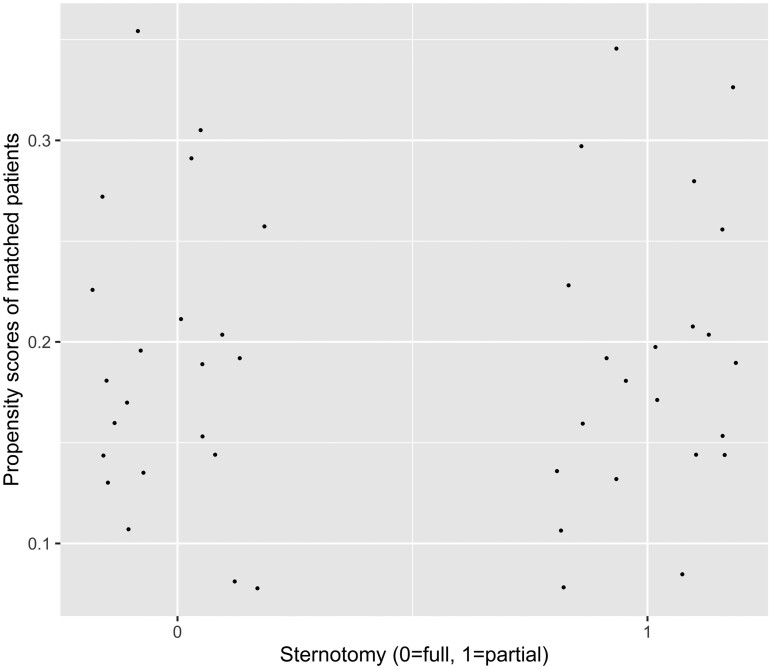
Jitter plot of between-group propensity score distribution in matched patients.

**Figure 2:**
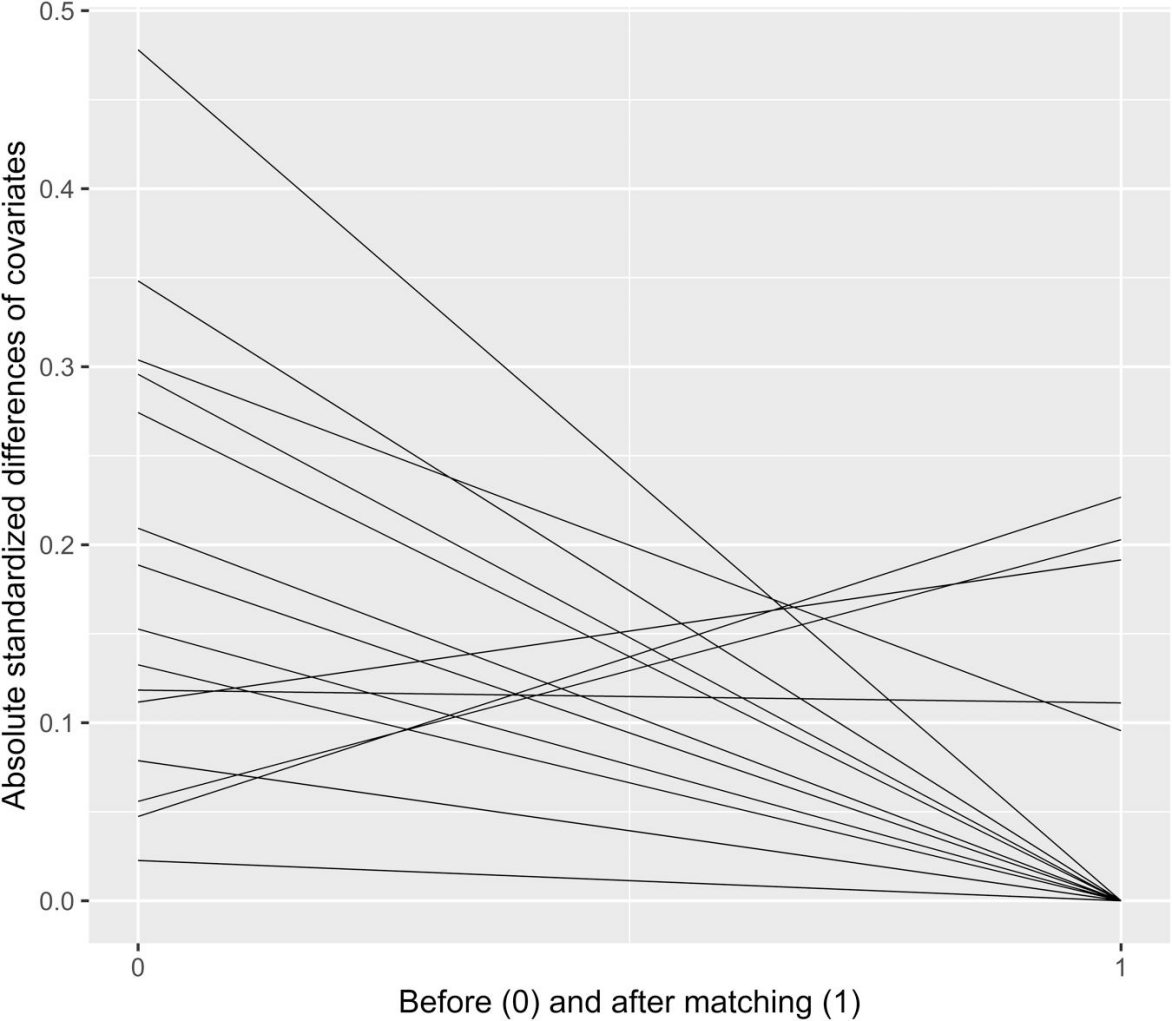
Line plot of absolute standardized differences of covariates before and after propensity score matching.

A total of 161 patients met the inclusion criteria, 22 with PUS and 139 with FS. Propensity score matching yielded a total of 44 patients or 22 patient pairs (PUS: *n* = 22, FS: *n* = 22) with an overall balanced distribution of propensity scores and covariates between the compared groups, as shown in the jitter plot of between-group propensity score distribution in matched patients (Fig. 1), and the line plot of absolute standardized differences of covariates before and after propensity score matching (Fig. 2). There was no evidence that PUS affected cardiopulmonary bypass [108 (67–119) min vs 113 (87–148), *P* = 0.154; PUS vs FS] and circulatory arrest duration [9 (7–10) min vs 9 (8–13), *P* = 0.264; PUS vs FS]. There was a reduced cross-clamp duration in the PUS group [88 (58–96) vs 92 (71–122) min, *P* = 0.032]. Temperature during circulatory arrest was higher in the PUS group [34 (34–36) vs 33 (32–34)°C, *P* = 0.031]. In the PUS group, 2 patients underwent valve-sparing aortic root replacement with reimplantation of the aortic valve (Tirone David procedure) and 1 valve-sparing aortic replacement with root remodelling (Yacoub procedure). The rest of the patients in the PUS group underwent aortic root and aortic valve replacement with reimplantation of the coronary arteries, using a biological conduit in 18 (Medtronic Freestyle prosthesis; Medtronic plc, Minneapolis, MN, USA) and a mechanical in 1 patient (Abbott Masters prosthesis; Abbott, Abbott Park, IL, USA). The preoperative and intraoperative patient characteristics of the unmatched and matched population are presented in Tables 1 and 2.

**Table 2:** Intraoperative characteristics of the study population

	Unmatched patients, *n* = 161			Matched patients, *n* = 44		
	Full sternotomy, *n* = 139	Partial sternotomy, *n* = 22	SD	Full sternotomy, *n* = 22	Partial sternotomy, *n *= 22	SD
CPB duration (min)	114 (83–136)	108 (67–119)	0.416	113 (87–148)	108 (67–119)	0.438
Aortic cross-clamping duration (min)	89 (67–107)	88 (58–96)	0.389	92 (71–122)	88 (58–96)	0.622
Circulatory arrest duration (min)	10 (8–13)	9 (7–10)	0.593	9 (8–13)	9 (7–10)	0.43
Temperature during circulatory arrest (°C)	32 (30–34)	34 (34–36)	1.482	33 (32–34)	34 (34–36)	0.991
Aortic valve reconstruction	26 (18.7)	3 (13.6)	0.138	8 (36.4)	3 (13.6)	0.544
Aortic valve reimplantation (Tirone David procedure)	25 (18)	2 (9.1)	0.262	8 (36.4)	2 (9.1)	0.688
Root remodelling (Yacoub procedure)	1 (0.7)	1 (4.5)	0.241	0	1 (4.5)	0.309
Bipolar pulmonary vein isolation	15 (10.8)	1 (4.5)	0.236	1 (4.5)	1 (4.5)	0
Left atrial appendage occlusion	16 (11.5)	0	0.51	1 (4.5)	0	0.309
Patent foramen ovale closure	4 (2.9)	1 (4.5)	0.088	1 (4.5)	1 (4.5)	0
Subaortic septal myomectomy	4 (2.9)	1 (4.5)	0.088	1 (4.5)	1 (4.5)	0

Continuous variables are reported as median (interquartile range), and categorical variables as counts and percentages, *n* (%).

CPB: cardiopulmonary bypass; SD: standardized difference.

### Postoperative outcomes

The postoperative outcomes are presented in Table 3. Perioperative mortality and morbidity were low, with no in-hospital mortality in the PUS group [0 vs 1(4.5%), *P* > 0.999], and absence of CVIs in both groups. Red blood cell concentrate, fresh frozen plasma and platelet concentrate transfusion were comparable between the 2 groups. There was no evidence that PUS affected re-exploration for bleeding [1 (4.8%) vs 1 (4.8%), *P* > 0.999; PUS vs FS], intensive care unit stay [1 (1–1.5) vs 1 (1–2.1) days, *P* = 0.750; PUS vs FS] and postoperative hospital stay [9 (7–10) vs 8 (7–11), *P* = 0.661; PUS vs FS]. Postoperative echocardiography revealed no between-group differences in left ventricular ejection fraction, mean and peak aortic transvalvular pressure gradient and aortic regurgitation rates.

**Table 3:** Postoperative characteristics of the study population

	Unmatched patients, *n* = 161			Matched patients, *n* = 44		
	Full sternotomy, *n* = 139	Partial sternotomy, *n* = 22	*P*-value	Full sternotomy, *n* = 22	Partial sternotomy, *n* = 22	*P*-value
Renal replacement therapy	3 (2.2)	1 (4.8)	0.445	1 (4.8)	1 (4.8)	>0.999
Rethoracotomy for bleeding or tamponade	3 (2.2)	1 (4.8)	0.443	1 (4.8)	1 (4.8)	>0.999
Postoperative cerebrovascular insult	5 (3.8)	0	>0.999	0	0	NA^a^
Intubation duration (hours)	5 (3–8)	4 (2.5–6)	0.089	5 (3–6.7)	4 (2.5–6)	0.262
ICU stay (days)	1 (1–2)	1 (1–1.5)	0.639	1 (1–2.1)	1 (1–1.5)	0.750
Postoperative stay (days)	9 (8–12)	9 (7–10)	0.372	8 (7–11)	9 (7–10)	0.661
Total RBCC transfusion (units)	0.3 (1.3)	0.5 (2.1)	0.719	0.4 (1.7)	0.5 (2.1)	>0.999
Total FFP transfusion (units)	0.3 (1.3)	0.09 (0.4)	0.441	0.4 (1.7)	0.09 (0.4)	0.414
Total PLC transfusion (units)	0.1 (0.3)	0.09 (0.2)	0.929	0.2 (0.6)	0.09 (0.2)	0.234
Postoperative myocardial infarction	2 (1.6)	1 (5)	0.353	1 (5.6)	1 (5)	>0.999
Postoperative LVEF (%)	55 (47–60)	55 (46–59)	0.517	55 (50–60)	55 (46–59)	0.551
Postoperative mean AV gradient (mmHg)	5 (4–6)	7 (5–11)	0.003	6 (4–8)	7 (5–11)	0.521
Postoperative peak AV gradient (mmHg)	9 (7–12)	14 (10–21)	0.002	11 (9–17)	14 (10–21)	0.246
Postoperative significant AR	0	0	N/A^a^	0	0	NA^a^
Death	2 (1.4)	0	>0.999	1 (4.5)	0	>0.999

Continuous variables are reported as mean (standard deviation) or median (interquartile range), and categorical variables as counts and percentages, *n* (%).

a No statistics were computed because the variable was a constant.

AR: aortic regurgitation; AV: aortic valve; FFP: fresh frozen plasma; ICU: intensive care unit; LVEF: left ventricular ejection fraction; NA: not available; PLC: platelet concentrate; RBCC: red blood cell concentrate.

### Learning curve assessment

A total of 4 surgeons performed the procedures in the PUS group. CUSUM charts have shown consistently low cross-clamping and circulatory arrest duration for the 2 most experienced surgeons who performed 20 of the procedures in the PUS group (10 each).

## DISCUSSION

The surgical management of complex aortic pathologies involving the aortic root, ascending aorta and aortic arch remains challenging in cardiac surgery. FS represents the gold standard for the surgical approach to such pathologies in most cardiac surgery departments [1, 2]. PUS represents less invasive access, allowing the concomitant management of aortic root, ascending aorta and proximal aortic arch disease; however, the reduced exposure might increase the difficulty of the procedure; thus, it has been used by experienced surgeons [10, 11]. Occasional series have reported the feasibility of performing isolated aortic root or ascending and hemiarch replacement via PUS [3–5]. The current study compared the early postoperative results of patients undergoing aortic root surgery with concomitant ascending aorta and hemiarch replacement via PUS versus FS, using propensity score matching.

The surgical procedures, including challenging aortic root reconstructions, were successful in all PUS patients, with no need for conversion to FS. Current perioperative results can be considered satisfactory and do not reveal limitations of PUS over FS. CBP and circulatory arrest times were comparable with patients who underwent aortic root, ascending aorta and hemiarch replacement operated via FS. Moreover, there was a reduced aortic cross-clamp duration via PUS.

To the best of our knowledge, no evidence regarding the perioperative outcome in less invasive aortic root surgery with concomitant ascending aorta and hemiarch replacement through PUS exists. Furthermore, previous studies are heterogeneous concerning their patient population, and a control group is usually absent.

In their work, Tabata *et al.* [10] reported that the PUS approach is safe and feasible for ascending aortic and hemiarch surgery, presenting excellent early outcomes in 128 consecutive patients. Byrne *et al.* [5] reported favourable outcomes of a less invasive approach via PUS to treat aortic root and complex ascending aortic pathologies. Our results underline these findings in the setting of concomitant aortic root, ascending aorta and proximal hemiarch surgery, with no in-hospital mortality and CVIs in the PUS group. Of note, there was no evidence of benefit regarding perioperative morbidity or intensive care unit and hospital stay for PUS.

Previous studies revealed an incidence of permanent neurological disorders following aortic arch surgery between 5% and 10% [12–16]. No permanent neurological disorders were detected in our preliminary series of 24 patients. Nevertheless, the correlation between PUS and these findings is not certainly causative, and the patient cohort is too small to draw any definite conclusion. However, this excellent neurological outcome aligns with previously published data from more extended series undergoing aortic arch surgery during selective antegrade cerebral perfusion in mild systemic hypothermia [17].

In a meta-analysis of studies using PUS as an approach for surgery either for aortic root or ascending aorta and proximal aortic arch, Perrotta and Lentini [11] highlighted that PUS could be used more liberally in the future, offering greater benefits to cardiac surgical patients. Motivated by this conclusion, we initiated our less invasive program for the concomitant surgery of aortic root, ascending aorta and proximal arch in 2019. Of note, almost all PUS procedures in our series were performed by 2 senior surgeons with constant exposure to the field of aortic root and thoracic aortic surgery, as well as several years of experience with left-sided ‘L’-shaped PUS in numerous patients. For these experienced surgeons, CUSUM chart analysis showed no learning curve for PUS, with consistently low cross-clamp and circulatory arrest duration. Encouraged by this proof of concept, younger surgeons are now being trained in this approach, considering that PUS offers less surgical trauma, better rib cage stability and improved cosmetic results.

In our cohort, PUS for concomitant aortic root, ascending aorta and hemiarch replacement was introduced only towards the end of the study period, in 2019, after accumulating experience with minimally invasive approaches and PUS over the previous years in simpler cases. Due to the PUS approach in our cohort being used only for concomitant procedures limited to the aortic root, ascending aorta and hemiarch, patients undergoing other major cardiac surgery were excluded from the analysis. This way, patients in the PUS and FS groups underwent the same major cardiac procedure, removing any possible effect of other concomitant major cardiac surgery on postoperative outcomes.

### Limitations

In the present study, some limitations must also be considered. Being a retrospective study, many biases inherently associated with this study type could not be excluded. Furthermore, the number of patients analysed was unavoidably small, as the present report represents our initial experience in less invasive concomitant aortic root, ascending aorta and aortic arch surgery. Finally, the majority of procedures in the PUS group were performed by 2 surgeons with great experience in aortic root and thoracic aortic surgery, as well as the PUS approach; therefore, the results presented in the current manuscript might not be generalizable.

## CONCLUSION

In summary, our initial experience suggests that less invasive aortic root, ascending aorta and hemiarch replacement via PUS can be performed safely with low early morbidity and mortality, comparable to that of FS, by surgeons with previous experience in aortic root and thoracic aortic surgery, as well as the PUS approach. Advantages for the patient are reduced surgical trauma, improved cosmetic results and—presumably—less pain.

## FUNDING

None declared.

**Conflict of interest:** none declared.

## Data Availability

The data supporting this study’s findings are available from the corresponding author upon reasonable request.

## Notes

Presented at the Annual Meeting of the European Association for Cardio-Thoracic Surgery, Vienna, Austria, 5 October 2023.

## ABBREVIATIONS

CUSUM: Cumulative sum
CVI: Cerebrovascular insult
EuroSCORE II: European System for Cardiac Operative Risk Evaluation II
FS: Full sternotomy
NYHA: New York Heart Association
PUS: Partial upper sternotomy
